# Descriptive Epidemiology of Kawasaki Disease in Japan, 2011–2012: From the Results of the 22nd Nationwide Survey

**DOI:** 10.2188/jea.JE20140089

**Published:** 2015-03-05

**Authors:** Nobuko Makino, Yosikazu Nakamura, Mayumi Yashiro, Ryusuke Ae, Satoshi Tsuboi, Yasuko Aoyama, Takao Kojo, Ritei Uehara, Kazuhiko Kotani, Hiroshi Yanagawa

**Affiliations:** Department of Public Health, Jichi Medical University, Shimotsuke, Tochigi, Japan; 自治医科大学地域医療学センター公衆衛生学部門

**Keywords:** mucocutaneous lymph node syndrome, incidence, cardiovascular disease, intravenous immunoglobulin, epidemiology

## Abstract

**Background:**

The number of patients and incidence rate of Kawasaki disease (KD) are increasing in Japan. We have therefore characterized the latest epidemiological information on KD.

**Methods:**

The 22nd nationwide survey of KD, which targeted patients diagnosed with KD in 2011 and 2012, was conducted in 2013 and included a total of 1983 departments and hospitals. In order to report on all patients with KD during the 2 survey years, we targeted hospitals of 100 beds or more with pediatric departments, or specialized pediatric hospitals.

**Results:**

From a total of 1420 hospitals and departments (71.6% response rate), 26 691 KD patients were reported (12 774 in 2011 and 13 917 in 2012; 15 442 males and 11 249 females). The annual incidence rates were 243.1 per 100 000 population aged 0 to 4 years in 2011 and 264.8 in 2012. The number of cases of KD recorded in 2012 was the highest ever reported in Japan. The incidence rate of complete cases was also the highest ever reported in Japan and contributed to the increase in the rate of total cases in recent years. The number of patients diagnosed per month peaked in January, and additional peaks were noted during summer months, although these peaks were lower than those seen in winter. Age-specific incidence rate showed a monomodal distribution with a peak in the latter half of the year in which patients were born.

**Conclusions:**

The number of patients and the incidence rate of KD in Japan continue to increase. A similar trend has also been seen for patients with complete KD.

## Abstract (ja)

**背景：:**

日本では川崎病の患者数、罹患率ともに増加の一途をたどっている。今回、川崎病の最新の疫学データを解析した。

**方法：:**

第22回川崎病全国調査を2013年に行った。2011年および2012年に川崎病を診断した1983施設を対象とした。小児科を標榜する100床以上の病院、および小児病院を対象とし、２年間の調査期間中に川崎病と診断されたすべての患者の報告を求めた。

**結果：:**

1420施設(回収率71.6%)から, 26,691人(2011年12,774人、2012年13,917人)の川崎病患者が報告された。男15,442人、女11,249人であった。0-4歳人口10万対罹患率は2011年243.1、2012年264.8であり、2012年の患者数は日本で報告されたものの中では最高であった。2012年の定型例の罹患率も日本で報告されたものの中で最高であり、近年の罹患率の増加に寄与した。月別推移では1月に患者数のピークがあり、夏にも1月に比べると低いピークがあった。年齢別罹患率では月齢6か月以内にピークを持つ一峰性の分布が見られた。

**結論：:**

川崎病の患者数および罹患率は日本においては増加し続けている。定型例においても同様の傾向が認められた。

## INTRODUCTION

Almost half a century has passed since Dr. Kawasaki first reported on 50 patients with Kawasaki disease (KD) in 1967 (http://www.jskd.jp/info/pdf/kawasaki.pdf), and since then, approximately 300 000 patients have been registered in Japan.^1^ However, the etiology of KD is still unknown. This disease affects infants and toddlers, and it causes systemic vasculitis.^2^^,^^3^ The vasculitis mainly attacks coronary arteries, and cardiac sequelae, such as coronary aneurysms and coronary insufficiencies, are some of the more serious outcomes of this disease.^4^^–^^8^ Fortunately, the proportion of patients with cardiac sequelae has decreased to less than 5%, down from 15%–20% approximately two decades ago. However, KD is still the leading cause of acquired heart disease in children, not only in Japan but also in European countries and the United States.^9^^,^^10^

Up to 2010, we conducted 21 nationwide epidemiologic surveys of KD.^1^ Previous nationwide surveys have revealed that the number of patients and incidence rate of this disease have been increasing yearly. As the etiology of this disease remains unknown, the reason for this increase is also unclear. Thus, surveillance of trends related to disease occurrence should be conducted.

Here, we describe the results of the 22nd nationwide survey of KD in Japan, which targeted KD diagnosed in 2011 and 2012.

## METHODS

We conducted a retrospective survey of patients with KD who visited target hospitals for treatment of acute KD during the two-year period from January 2011 through December 2012. This survey was conducted in almost the same manner as the 21st nationwide survey of KD.^1^

Participating medical facilities were hospitals specializing in pediatrics and hospitals with a total of 100 or more beds and a pediatric department. These criteria have been used since the first nationwide survey in 1970.^11^ Questionnaires (http://www.jichi.ac.jp/dph/kawasakibyou/20100715/kawasaki21final20100715.pdf, in Japanese) and diagnostic guidelines prepared by the Japan Kawasaki Disease Research Committee^12^ were sent by mail to administrators in charge of the pediatric department of their respective hospitals in January 2013. The prepared list of hospitals for the survey was based on the Listing of Hospitals 2003–2004 compiled by the Committee on Studies of Health Policies of Japan’s Ministry of Health, Labour and Welfare, and was revised using newly received information. A total of 2006 facilities met the conditions for inclusion. Patient information requested on the questionnaire was: name (initials only); address (municipality); sex; date of birth; date and day of first hospital visit for illness; diagnosis (typical definite, atypical definite, or incomplete); receipt of intravenous immunoglobulin (IVIG) therapy; IVIG resistance status; receipt of additional therapy, if conducted (additional IVIG therapy, steroids, infliximab, immunosuppressive agents, and plasmapheresis); recurrences; history of KD among patient’s siblings and parents; cardiac lesions (at the first visit, acute phase, and sequelae); and blood tests (white blood cell count, platelet count, albumin level, and C-reactive protein level). We classified typical definite cases (patients with five or six of the six symptoms specified in guidelines for the diagnosis of KD) and atypical definite cases (patients with four of the six diagnostic symptoms who had a coronary artery aneurysm, as confirmed by coronary angiography or two-dimensional echocardiography in the course of treatment) as complete cases. We classified patients who did not satisfy the diagnostic criteria for typical definite cases or atypical definite cases but were suspected of having KD by the pediatricians reporting the cases as incomplete type cases. Acute cardiac lesions were defined as those that developed within one month of the onset (acute lesions); cardiac sequelae were defined as those that persisted beyond one month after the onset. Almost all patients were diagnosed on the basis of two-dimensional echocardiography. After checking for possible inconsistencies on the questionnaires, the forms were sent back to the respondents to correct any errors. The incidence rates (reported as rate per 100 000 children aged 0 to 4 years) were based on the population data used in the vital statistics of Japan.^13^ The Bioethics Committee for Epidemiologic Research at Jichi Medical University approved this survey in advance (August 22, 2012; No. 12-18).

## RESULTS

Of the 2006 hospitals asked to participate in the survey, 23 reported that the pediatric department had been discontinued or that the hospital itself had fallen into disuse. Of the 1983 eligible hospitals receiving the invitation letter, 1420 (71.6%) returned the questionnaire. A total of 26 691 patients (12 774 in 2011 and 13 917 in 2012) were reported, including 15 442 male patients (7406 in 2011 and 8036 in 2012) and 11 249 female patients (5368 in 2011 and 5881 in 2012). The average annual incidence rate for the 2-year observation was 254.0 per 100 000 children aged 0 to 4 years (286.9 for males and 219.4 for females). The average annual incidence was 243.1 in 2011 and 264.8 in 2012, the highest ever reported in Japan.

**Figure 1.**
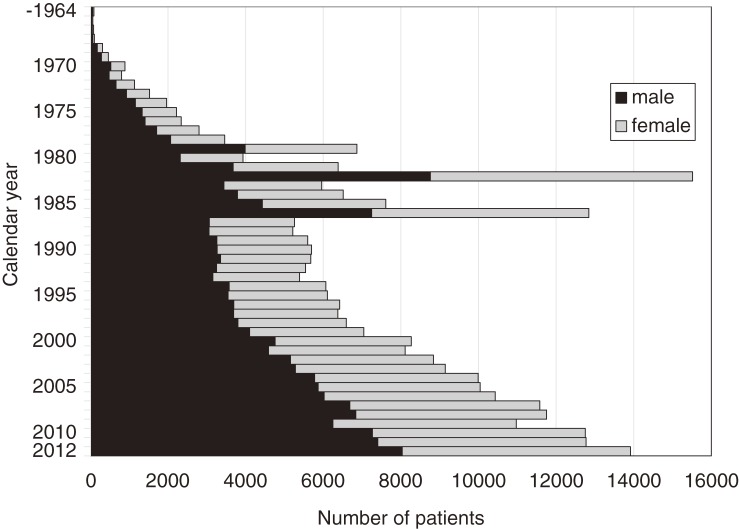
Number of patients with Kawasaki disease in Japan, by calendar year and sex

**Figure 2.**
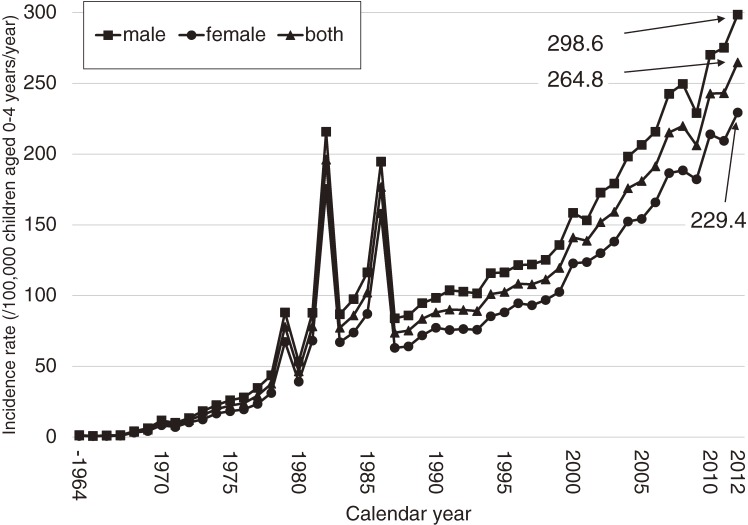
Incidence rate of Kawasaki disease in Japan, by calendar year and sex

**Figure 3.**
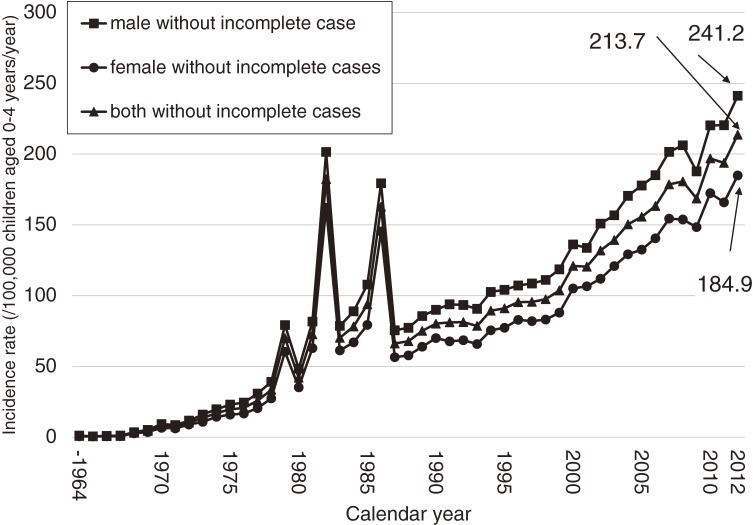
Incidence rate of complete Kawasaki disease in Japan, by calendar year and sex

**Figure 4.**
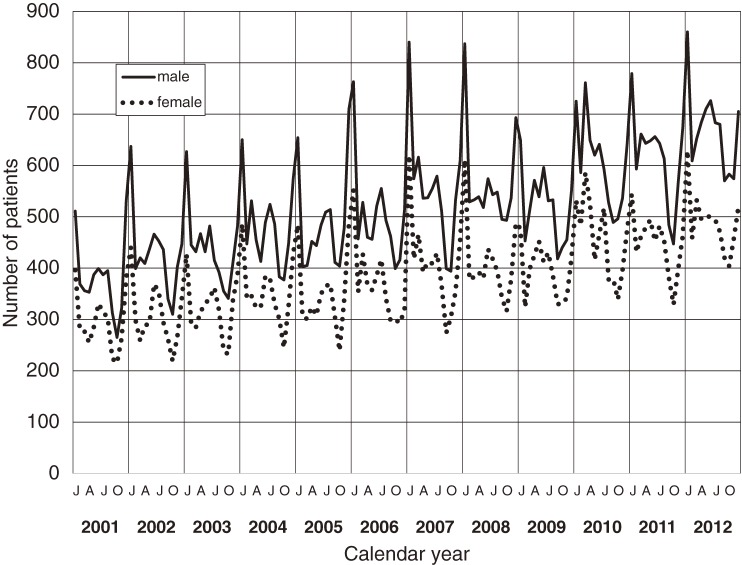
Number of patients with Kawasaki disease in Japan from 2001 through 2012, by month and sex

**Figure 5.**
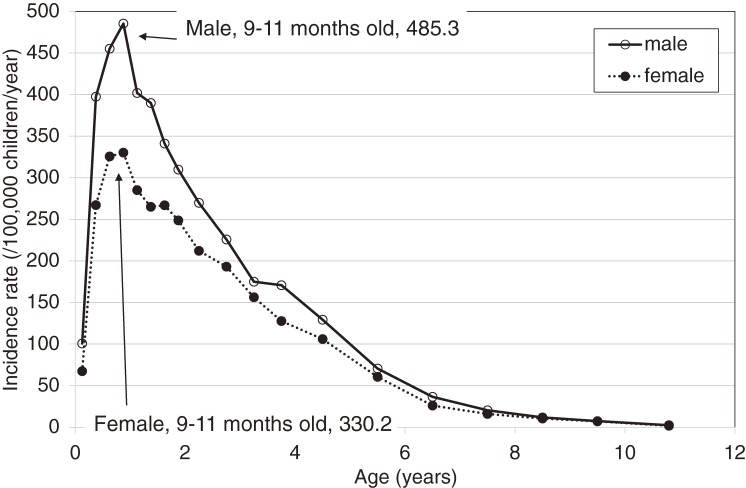
Age-specific incidence rate of Kawasaki disease from 2011 through 2012, by sex

The annual number of patients with KD and the incidence rates are shown in Figures 1 and 2. In 1979, 1982, and 1986, there were 3 large nationwide epidemics of the disease in Japan, as previously reported. Since then, no national epidemics have been observed; however, the number of patients with KD began to increase in the mid-1990s. The incidence rate has increased continuously since then, reaching 264.8 in 2012. This was the first time that the annual incidence rate has been higher than 250, exceeding the rates observed in 1979, 1982, and 1986, when nationwide epidemics occurred. Figure 3 shows the incidence rate of complete KD in the 22 nationwide surveys. It revealed that rate of complete KD has increased year by year (reaching 241.2 for males and 184.9 for females in 2012). Figure 4 shows the number of patients by month for the 6 most recent national surveys (17th to 22nd). Seasonal variations in the 21st survey differed slightly from those seen in previous surveys. Within the basic pattern of seasonal variation, there is a maximum peak in winter, and a slightly lower peak in summer. The peaks for the winters of 2009 and 2010 were lower than usual. However, seasonal patterns for 2012 were similar to those seen for the 17th–20th surveys. The peak in January 2012 was the highest ever reported in Japan. Figure 5 shows the age-specific incidence rates by sex. The incidence rate was highest in children aged 9 to 11 months. After this period, it decreased gradually with age.

There were no regional differences in the incidence rate of KD. Of the 26 691 patients who were reported, 20 915 (78.4%) were typical definite cases, and 486 (1.8%) were atypical definite cases. Therefore, 21 401 (80.2%) were complete cases, while 5274 (19.8%) were incomplete cases and 16 were unknown. Of the 5274 incomplete cases, 3575 (67.8%) had 4 of the 6 principal symptoms, 1321 (25.0%) had 3 symptoms, 311 (5.9%) had 2 symptoms, 41 (0.8%) had 1 symptom, and 26 were unknown. The number of patients with 1 or more siblings affected by KD was 408 (1.5%), and 273 (0.89%) patients had at least 1 parent with a history of KD. There were 946 (3.5%) cases of recurrence. Of the 26 691 patients reported, 4 patients (all male; two aged 0 to 5 months, one aged 1 year, and one aged 5 years) died.

At their first visit, 1241 (4.6%) patients had 1 or more cardiac lesions: 11 (0.04%) had giant coronary aneurysms, 66 (0.25%) had coronary aneurysms less than 8 mm in diameter, 957 (3.6%) had coronary dilatations, 3 (0.01%) had coronary stenoses, and 244 (0.91%) had valvular lesions (the sum of patients exceeds the total number of patients with cardiac lesions as some patients had more than one of these symptoms). During the acute phase, 2487 (9.3%) patients had 1 or more cardiac lesions: 47 (0.18%) had giant coronary aneurysms, 244 (0.91%) had coronary aneurysms less than 8 mm in diameter, 1866 (6.99%) had coronary dilatations, 5 (0.02%) had coronary stenoses, 1 (0.004%) had a myocardial infarction, and 443 (1.7%) had valvular lesions (the sum of patients exceeds the total number of patients with cardiac lesions as some patients had more than one of these symptoms). A total of 754 patients (2.8%) had cardiac sequelae 1 month after the onset of KD: 47 (0.18%) had giant coronary aneurysms, 191 (0.72%) had coronary aneurysms less than 8 mm in diameter, 466 (1.8%) had coronary dilatations, 6 (0.02%) had coronary stenoses, 1 (0.004%) had a myocardial infarction, and 98 (0.37%) had valvular lesions (the sum of percentages exceeds 100% as some patients underwent more than one treatment type). Furthermore, cardiac abnormalities were more prevalent in males (878 at their first visit, 1768 during the acute phase, and 571 with sequelae) than in females (403 at their first visit, 838 during the acute phase, and 238 with sequelae).

Of all patients, 24 346 (91.2%) received IVIG therapy. Of these, 20 954 (86.1%) underwent IVIG therapy in the 6 days after the onset of disease and 1279 (5.3%) received steroid therapy with IVIG therapy. However, 4150 (17.0%) of the 24 346 patients treated with IVIG did not respond to treatment (ie, fever lasted despite treatment), and 3798 of these (91.5% of non-responders) received additional IVIG therapy: 1245 (30.0%) with steroids, 179 (4.31%) with infliximab, 155 (3.73%) with immunosuppressants, and 93 (2.24%) with plasmapheresis (the sum of percentages exceeds 100% as some patients underwent more than one treatment type).

Upon analysis of the distribution of leukocyte levels by age at the first visit, the proportion of patients showing values greater than 10 000/µL tended to increase with age. In addition, the proportion of patients with platelet counts lower than 250 000/µL at first visit tended to increase with age, and those with counts higher than 400 000/µL tended to decrease with age. Regarding C-reactive protein levels by age at the first visit, the proportion of patients with levels in the range of 1–5 mg/dL and 5–10 mg/dL tended to decrease with age, while those showing a level higher than 15 mg/dL tended to increase with age. For these three factors and albumin level, there were no apparent differences observed between sexes.

## DISCUSSION

We have described the most recent epidemiological features of KD using our analysis of the results of the 22nd nationwide survey of KD in Japan. Since 1970, nationwide surveys have been carried out almost every 2 years.^1^^,^^14^^–^^19^ The number of patients and the incidence rate of this disease have increased rapidly since the mid-1990s. Due to the declining birth rate in Japan, the number of patients has increased less rapidly than the incidence rate of patients. However, the annual incidence rates in 2011 and 2012 were higher than those for years of nationwide epidemics (1979, 1982, and 1986), reaching 264.8 in 2012. The etiology of KD is unknown, and therefore, the reasons for these increases are also unclear. However these findings are very important, and attention should be paid to them. We need continued observation of the ongoing epidemiological features of KD in Japan.

After two reminders were sent, the response rate of the survey was 71.6%. Therefore, the actual number of patients with KD might be higher than that described by this study. Survey response rates have remained at approximately 70% over the past 10 years. In addition, we must remember that using the national population to calculate the incidence may result in underestimation of the true incidence, since patients may have received treatment in medical settings other than the ‘target hospitals’, such as small clinics not covered by the studied facilities. However, data suggest that the actual number of cases is likely to be within 10% of the value calculated from the survey, as discussed in a previous report.^20^

The seasonal epidemiologic features of KD in Japan observed in the latest survey are very similar to those seen before 2008 (Figure 4 and previous report^15^). In 2009, a new influenza strain (H1N1) was prevalent worldwide, including in Japan.^21^ It is possible that the influenza epidemic may have led to changes in the pattern of KD in 2009 and 2010, as the patterns observed were specific to these years. We compared the incidence rate of total cases and complete cases and found that the increase in total cases was due to the increase in complete cases in recent years.

One of the most serious problems in KD is cardiac sequelae, including coronary aneurysms, myocardial infarctions, and giant coronary aneurysms. Fortunately, the proportion of patients with coronary aneurysms and myocardial infarctions has decreased, from 6.0% for the 16th nationwide survey (1999–2000)^11^ to 2.8% for this survey. Furthermore, the proportion of patients with giant coronary artery aneurysms, the most serious sequela associated with KD, was also reduced substantially (0.40% in the 16th survey vs 0.18% in this survey). Advances in patient management during the acute phase are likely to have contributed to this improvement. Of the long-term outcomes of KD, the mortality rate with cardiac sequelae due to KD is the most important factor in KD management.^22^

A major feature of KD is seasonal variation in the number of patients affected. In Japan there is a maximum peak in January and a smaller peak in summer. The presence of seasonal variation supports the hypothesis that this disease is caused by an infectious agent. If KD is stimulated by several infectious agents of varying incidence in the peak season, there is the possibility that a number of different peaks are generated per year. In addition, the seasonality of KD differs by country and region,^23^^,^^24^ which might be due to the presence of different causative agents among these countries/areas. No geographic movement of KD was evident in data from the most recent nationwide survey. However, epidemiologic data from epidemic years (1979, 1982, and 1986) also supports the infectious agent hypothesis.^19^ On the other hand, increased likelihood of KD between parents and childen^25^ and siblings^26^ indicates a genetic cause. Therefore, we hypothesize that the onset of KD is involved with factors on the host side and infectious agents.

In conclusion, we have reported the result of the 22nd nationwide survey of KD in Japan. The number of patients and the incidence rate of KD continued to increase in 2011–2012. The incidence rate of complete KD also continued to increase in this period.

## ONLINE ONLY MATERIAL

Abstract in Japanese.
