# Supplementary material for: Descriptive Epidemiology of Kawasaki Disease in Japan, 2011–2012: From the Results of the 22nd Nationwide Survey
Source: J Epidemiol. 2015 Mar 5;25(3):239–45. doi: 10.2188/jea.JE20140089 (PMC4341001; doi:10.2188/jea.JE20140089)
Supplement: Abstract in Japanese. [file je-25-239-s001.pdf]

日本における川崎病の疫学像:2011-2012 年の全国調査結果

牧野伸子[1]、中村好一[1]、屋代真弓[1]、阿江竜介[1]、坪井　聡[1]、青山泰子[1]、古城隆雄[1]、上原里程[2]、小谷和彦[1]、柳川　洋[1]

1 自治医科大学地域医療学センター公衆衛生学部門

2 宇都宮市保健所

背景: 日本では川崎病の患者数、罹患率ともに増加の一途をたどっている。今回、川崎病の最新の疫学データを解析した。

方法: 第 22 回川崎病全国調査を 2013 年に行った。2011 年および 2012 年に川崎病を診断した 1983 施設を対象とした。小児科を標榜する 100 床以上の病院、および小児病院を対象とし、2年間の調査期間中に川崎病と診断されたすべての患者の報告を求めた。

結果: 1420 施設 (回収率 71.6%)から, 26,691 人(2011 年 12,774 人、2012 年 13,917 人)の川崎病患者が報告された。 男 15,442 人、女 11,249 人であった。 0-4 歳人口 10 万対罹患率は 2011 年 243.1、2012 年 264.8 であり、2012 年の患者数は日本で報告されたものの中では最高であった。2012 年の定型例の罹患率も日本で報告されたものの中で最高であり、近年の罹患率の増加に寄与した。 月別推移では 1 月に患者数のピークがあり、夏にも 1 月に比べると低いピークがあった。年齢別罹患率では月齢 6 か月以内にピークを持つ一峰性の分布が見られた。

結論: 川崎病の患者数および罹患率は日本においては増加し続けている。定型例においても同様の傾向が認められた。

キーワード: 皮膚粘膜リンパ腺症候群、罹患率、心血管疾患、免疫グロブリン静注療法、疫学
